# Single Nuclei Transcriptomics Reveals Obesity-Induced Endothelial and Neurovascular Dysfunction: Implications for Cognitive Decline

**DOI:** 10.3390/ijms252011169

**Published:** 2024-10-17

**Authors:** Dragan Milenkovic, Saivageethi Nuthikattu, Jennifer E. Norman, Amparo C. Villablanca

**Affiliations:** 1 Department of Nutrition, University of California, Davis, CA 95616, USA; 2 Department of Internal Medicine, Division of Cardiovascular Medicine, University of California, Davis, CA 95616, USA; snuthikattu@ucdavis.edu (S.N.); jenorman@ucdavis.edu (J.E.N.); avillablanca@ucdavis.edu (A.C.V.)

**Keywords:** single nuclei transcriptomics, obesity, hippocampus, neurovascular unit

## Abstract

Obesity confers risk for cardiovascular disease and vascular dementia. However, genomic alterations modulated by obesity in endothelial cells in the brain and their relationship to other neurovascular unit (NVU) cells are unknown. We performed single nuclei RNA sequencing (snRNAseq) of the NVU (endothelial cells, astrocytes, microglia, and neurons) from the hippocampus of obese (*ob*/*ob*) and wild-type (WT) male mice to characterize obesity-induced transcriptomic changes in a key brain memory center and assessed blood–brain barrier permeability (BBB) by gadolinium-enhanced magnetic resonance imaging (MRI). *Ob*/*ob* mice displayed obesity, hyperinsulinemia, and impaired glucose tolerance. snRNAseq profiled 14 distinct cell types and 32 clusters within the hippocampus of *ob*/*ob* and WT mice and uncovered differentially expressed genes (DEGs) in all NVU cell types, namely, 4462 in neurons, 1386 in astrocytes, 125 in endothelial cells, and 154 in microglia. Gene ontology analysis identified important biological processes such as angiogenesis in endothelial cells and synaptic trafficking in neurons. Cellular pathway analysis included focal adhesion and insulin signaling, which were common to all NVU cell types. Correlation analysis revealed significant positive correlations between endothelial cells and other NVU cell types. Differentially expressed long non-coding RNAs (lncRNAs) were observed in cells of the NVU-affecting pathways such as TNF and mTOR. BBB permeability showed a trend toward increased signal intensity in *ob*/*ob* mice. Taken together, our study provides in-depth insight into the molecular mechanisms underlying cognitive dysfunction in obesity and may have implications for therapeutic gene targeting.

## 1. Introduction

Obesity is a global pandemic [1] and the fifth leading cause of death worldwide [2]. The World Health Organization estimates nearly 2.8 million people with obesity die annually [1]. By 2030, the number of individuals with obesity is estimated to be 573 million [3]. Obesity is associated with various comorbidities, including cardiovascular disease, diabetes mellitus, hypertension, and stroke [4], and is also a key risk factor for several neurodegenerative diseases, including Alzheimer’s disease (AD) [5]. Individuals with obesity are also predisposed to dementia and impaired cognitive function, including short-term memory and learning [6].

The cerebral microcirculation plays an important role in neuronal function [7], and damage to cerebral blood vessels contributes to cognitive decline and dementia [8]. It has been shown that obesity can trigger changes in the cerebral vasculature [7] and promote neurovascular inflammation and oxidative stress, which cause cerebral hypoperfusion resulting in a disrupted blood–brain barrier (BBB) [9]. Accordingly, the National Institute on Aging’s AD + Alzheimer’s Disease-Related Dementias (ADRD) Research Implementation Milestone 2.B prioritizes research to “determine interrelationships among aging, cerebrovascular disease and risk factors, resilience factors, genetic variants, amyloid, tau, and neurodegeneration” [10].

The molecular pathways by which obesity affects the brain are being elucidated. Recent studies have shown modulated expression of a vast number of obesity-associated genes in the brain [11], including elevated expression of genes associated with inflammation and immunosenescence [12]. A microarray study of human brains identified a decrease in the expression of zinc transporter proteins, increasingly connected with the formation of senile plaques, in both AD and obesity [13]. In rodent models of obesity and AD, an increase in hippocampal expression of the inflammatory mediator inducible nitric oxide synthase (iNOS) [14,15] was observed. Furthermore, RNA sequencing analysis on the postmortem hypothalamus of individuals with obesity revealed differential expression of genes involved in metabolism, immunity, and inflammation [16]. However, this study only focused on whole brain tissue, making it challenging to gain insight for cell type-specific gene expression changes. Therefore, the molecular mechanisms of obesity-mediated cerebrovascular dysfunction and its association with neurodegeneration are not yet fully defined.

There is increasing data supporting the importance of the vascular effects of obesity as contributors to vascular dementia. The mechanisms may be partly driven by a reduction in hippocampal microvascular density and alterations in neurovascular coupling [17,18,19,20]. The neurovascular unit (NVU) is essential for BBB integrity and is composed of endothelial cells, glial cells (microglia and astrocytes), and neurons [21]. The NVU plays an important role in the pathogenesis of vascular dementia involving reduced cerebral blood flow, which leads to neuronal and glial cell damage, dysregulation of endothelial cells, and breakdown of the BBB [22]. Recently, single-cell RNA sequencing (scRNAseq) of high-fat diet-induced obesity in mice identified dysregulation of hippocampal microglial cells [23]. However, to date, no studies have utilized single nuclei RNA sequencing (snRNAseq) to explore the effects of obesity on coordinated gene expression in endothelial cells themselves or in the context of other hippocampal NVU cell types and their contributions to vascular dementia. In contrast to traditional bulk RNA sequencing methods, single-cell/nuclei RNA sequencing technology has the advantage of revealing cell-specific changes in expression of genes, relationships between different cell types, cellular function, and pathology of individual cells [24]. In scRNAseq, whole cells are isolated, and RNA is captured from the entire cell, whereas snRNAseq involves isolating only the nuclei and focusing on RNA within the nucleus [25]. snRNAseq has the additional advantage of working with complex and fragile brain tissue, potentially yielding more reliable and less biased results. Although scRNAseq and snRNAseq should theoretically yield comparable results when studying obesity-induced neurovascular dysregulation, the snRNAseq approach may provide more accurate insights into the nuclear gene expression alterations associated with this condition, primarily due to the sampling procedure.

Hence, in accordance with national research priorities, our study aims to better understand the mechanisms by which obesity induces genomic changes in the cells of the NVU and its relevance to the vascular effects of obesity as contributors to vascular dementia. We performed an integrative multiomics study using state-of-the-art single nuclei RNA sequencing of the NVU (endothelial cells, neurons, microglial cells, and astrocytes) from the hippocampus, an important brain memory center, of *ob*/*ob* mice [26,27]. We sought to identify the effect of obesity on transcriptomic changes in the hippocampal NVU characterized by differentially expressed protein-coding and non-coding genes and the pathways involved. We also performed functional assessments of BBB permeability by utilizing structural brain MRI imaging. Based on our previous studies [28,29,30,31,32,33], we hypothesized that *ob*/*ob* mice would exhibit cell-specific and common expression alterations in the gene expression profiles of the cells of the NVU and that the changes could be characterized by pathways and networks enriched for endothelial cell function, neuroinflammation/degeneration, and BBB breakdown.

## 2. Results

### 2.1. Single-Nuclei RNA Sequencing Identifies 14 Cell Types in the Hippocampus

**Figure 1:**
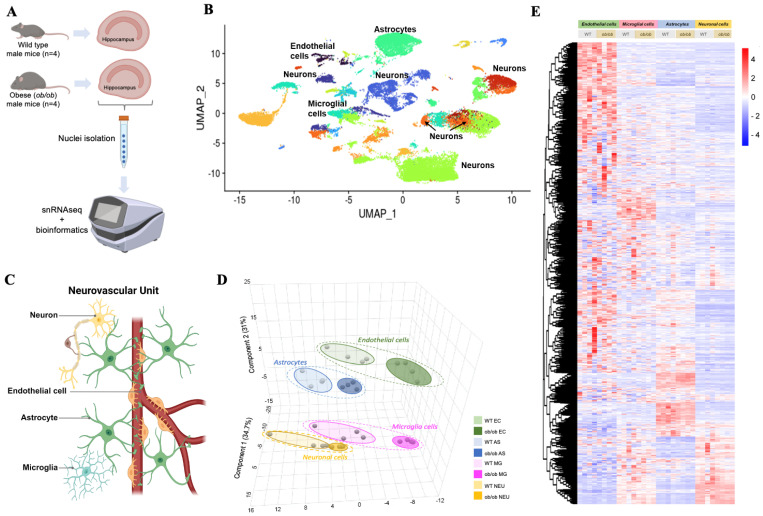
**Single nuclei RNA-sequencing identifies global genomic cell-specific changes in the hippocampus of obese mice.** (**A**) Overview of this study. Hippocampi were isolated from normal weight (wild type) and from obese (*ob*/*ob*) male mice (*n* = 4/genotype) at 18 weeks of age. Single nucleotide RNA sequencing was performed on isolated hippocampi, followed by in-depth bioinformatics analyses. (**B**) Uniform manifold approximation and projection (UMAP) showing the cell clusters in hippocampal cells in obese and normal-weight mice. (**C**) Schematic presentation of the major cells of the neurovascular unit (NVU) created in biorender.com (accessed on 27 February 2024). (**D**) Sparse Partial Least Squares Discriminant Analysis (sPLS-DA) performed using normalized global gene expression datasets from NVU cell types. (**E**) Heatmap of normalized gene expression, where the rows are expressed genes and the columns are the individual samples, grouped by cell types of NVU. Red denotes higher levels of gene expression, and blue denotes lower levels of gene expression, as indicated in the color bar.

Compared to WT mice, the *ob*/*ob* phenotype at 17–18 wks of age was characterized as obese, hypercholesterolemic, and hyperinsulinemic and had impaired glucose tolerance, though it was not hyperglycemic (Table S1). To assess the impact of obesity on the NVU transcriptome, we performed single nuclei RNA sequencing (snRNAseq) of the hippocampus from *ob*/*ob* and WT mice (Figure 1A). UMAP (Uniform Manifold Approximation and Projection) analysis revealed 14 cell types, which formed 32 clusters (Figure 1B). Table S2 lists the mean number of hippocampal cell types, including those that comprised the NVU.

### 2.2. Cell-Specific Gene Expression Alterations with Obesity in the NVU

First, we compared global gene expression profiles between the two genotypes in each of the four cell types of the NVU (Figure 1C). Sparse Partial Least Squares Discriminant Analysis (sPLS-DA) identified different global gene expression profiles between *ob*/*ob* and WT for each of the cell types of the NVU (Figure 1D). The top 10 genes for each NVU cell type driving the separation (as determined by variable importance projection scores) between *ob*/*ob* and WT mice can be found in Figure S1. Genes that were important in the separation for the NVU included *Nr1d1* and *Slc39a13* (endothelial cells), Ddc and Tppp (microglia), Stat5b and Fam214a (astrocytes), and Tnrc6b and Muc6 (neurons) (Figure S1). A heat map of expression profiles of genes showed that endothelial cells in *ob*/*ob* and WT mice present overall higher levels of gene expression when compared to other cell types of the NVU (Figure 1E).

**Figure 2:**
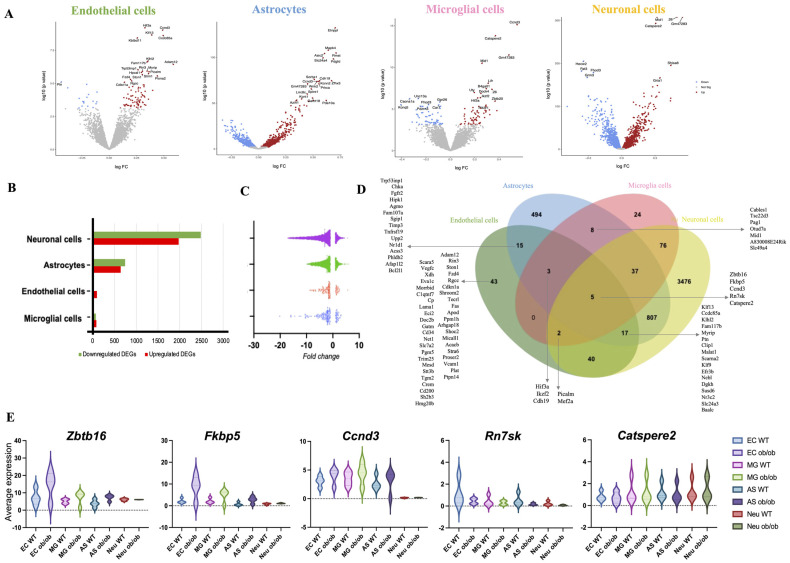
**Obesity significantly changes gene expression in endothelial, microglial, astrocytes, and neuronal cells of the hippocampus.** (**A**) Volcano plots show differential gene expression for *ob*/*ob* mice compared to control WT mice for the four cell types of NVU. Only significantly changed gene expressions (*p* < 0.06) are colored according to the direction of change, that is, blue for downregulation and red for upregulation of gene expression. (**B**) Bar plot showing the number of differentially expressed genes (DEGs) both downregulated (green) and upregulated (red) across the four cell types of the NVU. (**C**) Distribution of fold changes for significant DEGs between obese and normal-weight mice across NVU cell populations. (**D**) Venn diagram of DEGs in endothelial cells (EC), microglial cells (MG), astrocytes (AS), and neurons (NEU) showing overlaps between sets of identified genes. (**E**) Violin plots of the expression levels presented as the normalized counts of common DEGs across cell types of the NVU (EC, MG, AS, and NEU).

Next, we aimed to identify differentially expressed genes (DEGs) altered by obesity in the four cell types in the NVU. We identified that compared to WT, the *ob*/*ob* genotype significantly modulated the expression of 4462 DEGs in neurons, 1386 DEGs in astrocytes, 125 DEGs in endothelial cells, and 154 DEGs in microglial cells (Figure 2A,B and Supplementary Data S1). The observed fold changes in gene expressions varied from −10 to 5 (Figure 2C). Comparison of DEGs of NVU cells revealed only five DEGs in common (Figure 2D) and included Zbtb16, Fkbp5, Ccnd3, Rn7sk, and Catspere2; their expression levels are presented in Figure 2E. This observation is consistent with a cell-specific impact of obesity in the expression profiles of hippocampal NVU cells.

### 2.3. Functional Enrichment Analyses of DEGs of NVU Cells in Obesity

We then performed functional enrichment analyses of DEGs modulated by obesity in the NVU cell types. First, we performed a gene ontology (GO) analysis of DEGs to functionally classify the biological processes (BP) impacted by obesity. Among the most significantly over-represented GO BP terms are regulation of angiogenesis, cell adhesion, proliferation, migration, and actin cytoskeleton organization associated with endothelial DEGs (Figure S2A), while astrocyte DEGs were involved in cell substrate adhesion and Rho protein signal transduction (Figure S2B). Microglial DEGs were involved in actomyosin structure organization and cognition (Figure S2C), and neuronal DEGs were associated with vesicle-mediated transport in synapse and peptidyl-serine phosphorylation (Figure S2D). Cell junction assembly and small GTPase-mediated signal transduction were common to most cells of the NVU (endothelial cells, astrocytes, and neurons), as were dendrite development and synapse and cell projection organization (astrocytes, microglia, and neurons). Thus, most of the DEGs modulated by obesity impacted the cells of the NVU in functionally distinct patterns.

**Figure 3:**
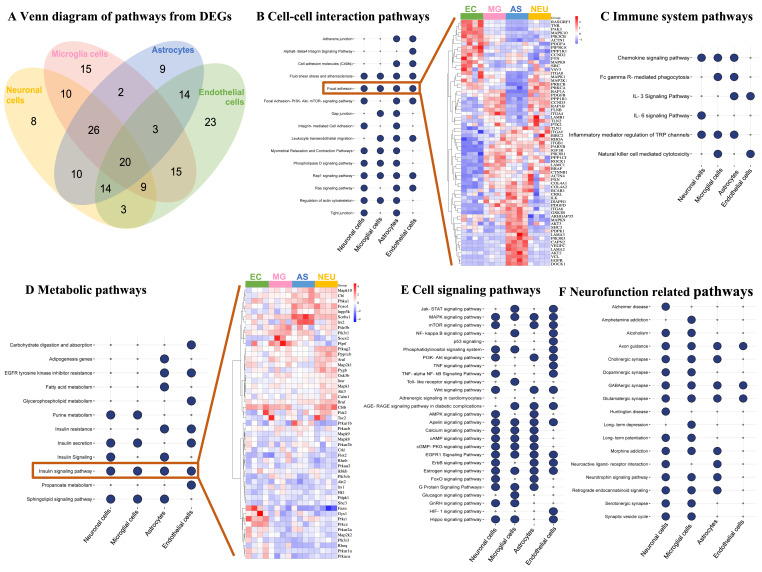
**Functional classification of DEGs identifies cell-specific and common major cellular pathways regulating cell–cell interactions, metabolism, immune system, cell signaling, and neurofunction across cells of the NVU.** (**A**) Venn diagram comparing identified significantly enriched pathways for the four cell types of the NVU with DEGs altered by obesity compared to normal-weight mice. (**B**) Dot plot of cell–cell interaction pathways regulated by DEGs in the NVU cell types. Significant pathways are presented with large blue circles. A heatmap representing expression levels of genes involved in focal adhesion across the NVU cells is presented on the right. (**C**) Dot plot of immune system pathways modulated by DEGs in the NVU cell types. (**D**) Dot plot of pathways of DEGs involved in the regulation of metabolism in the NVU cells. A heatmap representing expression levels of genes involved in the insulin signaling pathway across the NVU cell types is presented on the right. (**E**) Dot plot of pathways of DEGs involved in cell signaling in the NVU cells. (**F**) Dot plot of pathways of DEGs involved in the neurofunction regulation in the NVU cell types.

Subsequently, we identified enriched cellular pathways involving the DEGs of NVU cell types. Among the top 50 cellular pathways (FDR *p* < 0.05), NVU cell type-specific pathways included alpha6-beta4 integrin signaling (endothelial cells), glucagon signaling (microglia), fatty acid metabolism (astrocytes), and IL-6 signaling (neurons) (Figure 3A and Figure S3). However, 20 pathways were in common between the four NVU cell types. They included focal adhesion, axon guidance, Rap1, insulin, MAPK, and EGFR1 signaling.

We then classified cellular pathways into five functional categories as follows: cell–cell interaction (Figure 3B), immune system (Figure 3C), metabolic (Figure 3D), cell signaling (Figure 3E), and neurofunction-related (Figure 3F) pathways. Among cell–cell interaction pathways (Figure 3B), focal adhesion was common to all the NVU cell types. Using hierarchical clustering, comparison of expression profiles of genes involved in this pathway showed that Rasgrf1, Tnr, Pak3, Mapk10, Pdgfa, Pip5k1c, Ppp1r12c, Mapk8, and Src genes were upregulated by obesity in endothelial cells, whereas downregulated in the other cell types, suggesting that even though DEGs were involved in a similar cellular process, there was cell specificity leading to distinct expression profiles. Additionally, within the metabolic functional category, insulin signaling was common to all NVU cell types (Figure 3D). In contrast to genes involved in focal adhesion, the expression profiles of genes involved in insulin signaling were modulated by obesity in the same direction in all four NVU cell types. Taken together, these snRNAseq data suggest that obesity modulated the genomic profiles of NVU cells involved in the regulation of cell junctions, cell signaling, metabolic processes, and inflammation.

### 2.4. Gene Expression Profiles of Endothelial Cells Positively Correlate with Other Cell Types of the NVU

**Figure 4:**
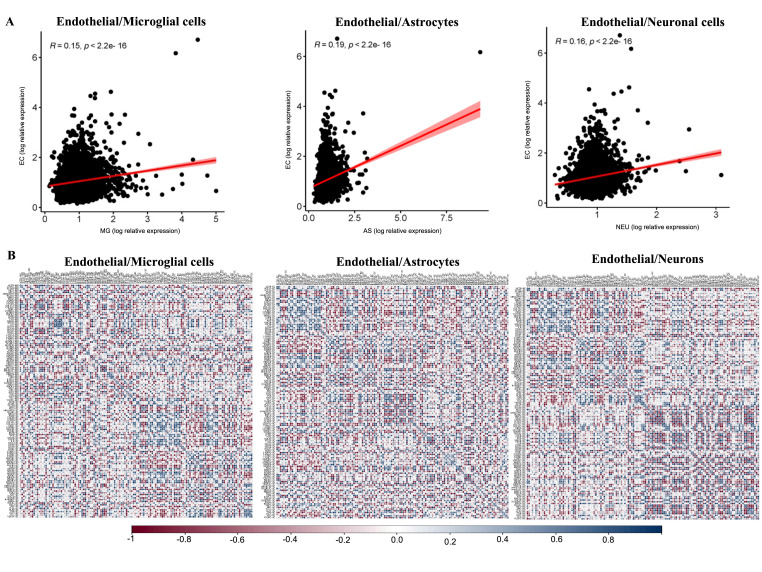
**Positive correlation of global gene expression changes by obesity in endothelial cells with other cell types of the hippocampal NVU.** (**A**) Scatter plots of genes showing significant (*p* < 0.05) positive correlation between endothelial cells and microglia cells (r = 0.15), endothelial cells and astrocytes (r = 0.19), and endothelial cells and neuronal cells (r = 0.16). (**B**) Gene–gene correlation matrices of genes identified as differentially expressed in endothelial cells and microglia cells, endothelial cells and astrocytes, and endothelial cells and neuronal cells. For each gene–gene correlation, positive correlation is presented in blue and negative correlation in red; significant (*p* < 0.05) correlations are presented with “*”.

As endothelial cells play a major role in the regulation of vascular permeability in the brain, we sought to assess how changes in the endothelial cells’ gene expression relate to gene expression in other cell types of the NVU. For this analysis, we performed correlations between the changes in the gene expression of endothelial cells with changes in the other NVU cells. There was a significant (*p* < 0.05) positive correlation between endothelial cells and microglia (r = 0.15), astrocytes (r = 0.19), and neurons (r = 0.16) (Figure 4A). We then performed correlation analysis between DEGs altered by obesity in endothelial cells with all of the detected genes in microglia, astrocytes, and neuronal cells (Figure 4B and Supplementary Data S2). We found that 25 DEGs in endothelial cells, each correlated with over 10 genes in astrocytes. Among these endothelial DEGs with the highest number of correlations with astrocyte genes were Fam117b, Afap1l2, Nr3c2, Gpcpd1, Malat1, Plat, or Clip1. Similarly, six endothelial DEGs presented correlations with 10 microglial genes, including Ikzf2, Parvb, Tacc1, Nr1d1, Micall1, and Clip1. Moreover, we observed that seven endothelial DEGs had significant correlation with 10 or more neuronal genes, including Rn7sk (correlated with 13 neuronal genes), Baalc, Eva1c, Fam117b, Arhgap18, Klhl2, and Herc4 (correlated with 10 neuronal genes).

Furthermore, since the focal adhesion pathway was identified as over-represented in all four NVU cells and is an important regulator of cell–cell interaction and permeability, we performed correlation analyses between genes altered by obesity in this pathway in any of these cell types. This analysis demonstrated that expression of seven endothelial genes correlated with expression of more than five genes in microglia, including Rock1, Itgav7, Ppp1r12c, and Lama3 (Figure S4). Expression of genes such as Vegfc, Ink, Mapk10, and Pdgfb in endothelial cells correlated with expression of six or more astrocyte genes. Pdpk1, Prkca, and Rhoa genes expressed in endothelial cells correlated with the expression of five neuronal genes. Finally, endothelial expression of Ppp1cb and Shc33 correlated with expression of both microglia and astrocyte genes, while endothelial expression of Itgb1 correlated with expression of both microglia and neuron genes. Taken together, these results indicate that changes in the expression of genes in endothelial cells induced by obesity may impact gene expression in other cells of the NVU, suggesting there may be NVU cellular cross-talk.

### 2.5. Cell-Specific Transcriptional and Post-Transcriptional Regulators of Gene Expression

The next step of our analyses was to detect transcriptional factors (TFs) whose activity could be upstream of the observed obesity-induced genomic alterations. A comparison of the top 20 TFs involved in the transcriptional regulation of DEGs identified TFs unique to each of the NVU cell types, including PPARG, POU5F1, GATA2 for endothelial cells; BACH2, NEUROG1, STAT1 for microglia; NFKB1, ASCL1, STAT3 in astrocytes; and LIN28, HIVEP2, and MEF2D in neurons (Figure S5). REST TF was the only common TF to all the four cell types of the NVU.

**Figure 5:**
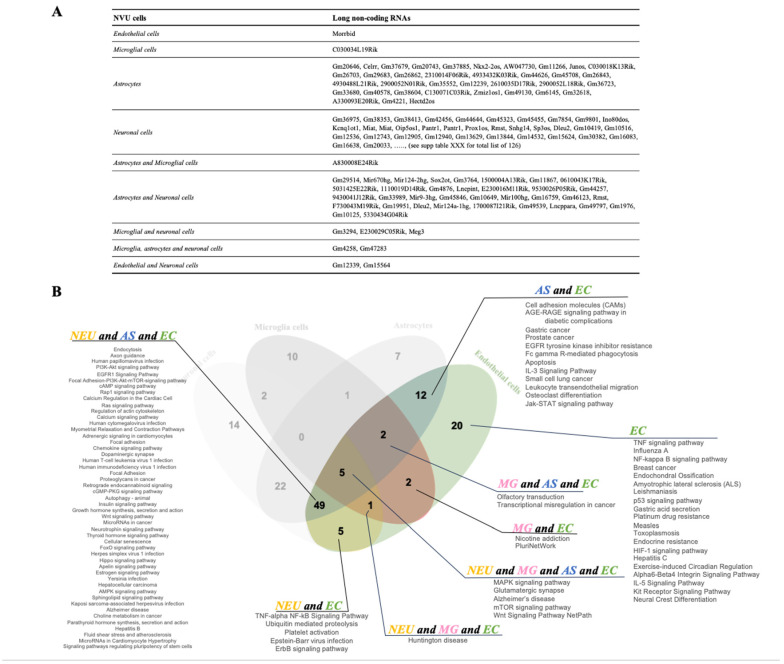
**Obesity induces cell-specific changes in the expression of long non-coding RNAs.** (**A**) Table with identified differentially expressed (DE) long non-coding RNAs (lncRNAs) specific to each cell type and in common among the cells of NVU. (**B**) Pathway enrichment analysis of target genes of the 20 most significant DE lncRNAs in the NVU cell types. The colored Venn diagram shows pathways specific to endothelial cells (EC) and in common to other NVU cell types (NEU: neuronal cells; AS: astrocytes; MG: microglia).

Together with transcriptional regulators, the expression of genes can also be modulated by post-transcriptional regulators. Indeed, our snRNAseq revealed that obesity can alter the expression of a number of long non-coding RNAs (lncRNAs) in the NVU (4 in endothelial cells, 7 in microglia, 75 in astrocytes, and 169 in neurons; Figure 5A). Cell-specific lncRNAs included Morrbid (endothelial cells), C030034L19Rik (microglia), C030018K13Rik (astrocytes), and Miat (neurons). Gm4258 and Gm47283 were common to astrocytes, microglia, and neurons, while Gm12339, Gm15564, and Malat1 lncRNAs were common to endothelial cells and neurons. These results suggest that, as is the case for protein-coding genes, obesity has a distinct and primarily cell-specific impact on both transcriptional and post-transcriptional regulation of cells of the NVU.

Next, we identified potential targets of the top 20 most significantly modified lncRNAs. We identified 341, 392, 700, and 1053 target genes for these top 20 differentially expressed (DE) lncRNAs in endothelial cells, microglia, astrocytes, and neurons, respectively. Comparison of DEGs and potential target genes of the top 20 DE lncRNAs for each NVU cell type showed that 0.5% to 5% of the lncRNA target genes were differentially regulated by obesity, showing that although not a primary process, lncRNA regulation of gene expression is partially responsible for the observed transcriptomic changes in obesity (Figure S6).

Furthermore, we then performed pathway analysis to identify potential biological processes affected by alteration in the expression of the top 20 lncRNAs. Comparison of cellular pathways regulated by the targets of the top 20 DE lncRNAs of hippocampal NVU cells revealed pathways unique to endothelial cells such as TNF, NFkB, HIF-1, and IL-5 signaling (Figure 5B and Figure S7). Five pathways were in common to all the NVU cell types, including Alzheimer’s disease, glutamatergic synapse, mTOR, MAPK, and Wnt signaling. Focal adhesion, Rap1 signaling, and regulation of the actin cytoskeleton were some of the pathways in common with endothelial cells, astrocytes, and neuron cells. Therefore, obesity modulates the expression of lncRNAs that results in the regulation of biologic processes important in neurovascular function such as cell–cell junctions, endothelial cell permeability, cell signaling, and neuronal synapses.

### 2.6. Prediction of Cell-Specific Clinical Diseases Associated with Gene Expression Alterations by Obesity in the NVU

**Figure 6:**
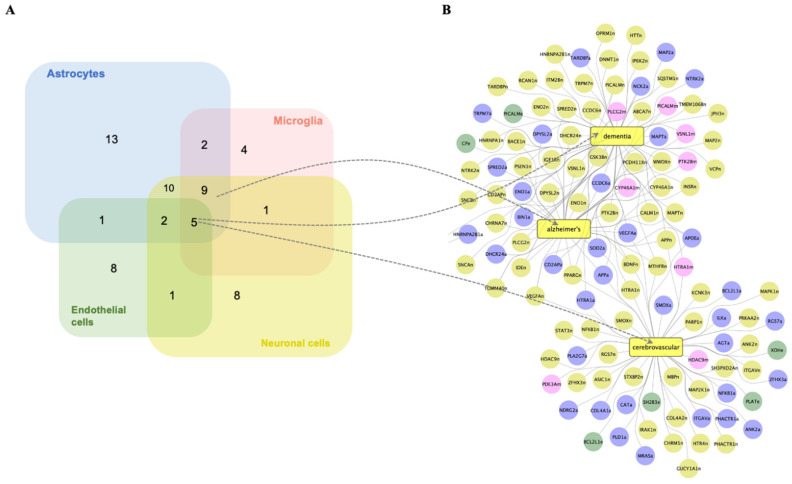
**Obesity modulates the expression of genes in NVU cell types associated with neurodegenerative diseases.** (**A**) Venn diagram showing the number of significant associations between DEGs of NVU cell types and neurodegenerative diseases. (**B**) Network representing interactions between genes differentially expressed by obesity in the NVU cell types with Alzheimer’s disease, cerebrovascular diseases, and dementia. DEGs in endothelial cells (e, green circles), astrocytes (a, blue circles), neuronal cells (n, yellow circles), and microglial cells (m, pink circles).

We identified neurodegenerative diseases associated with DEGs in the NVU cell types (Figure 6A). Dementia and cerebrovascular diseases were common diseases associated with DEGs of all the four NVU cell types, while Alzheimer’s disease (AD) was common to all NVU cell types except astrocytes. Next, we did network analysis of DEGs associated with AD, dementia, and cerebrovascular diseases for each of the NVU cell types (Figure 6B). Endothelial cell DEGs *Picalm* and *Cp* were associated with dementia, while *Plat* and *Sh2b3* were associated with cerebrovascular disease. Other NVU cell-type DEGs formed connections with both dementia and AD, including *Plcg2* (microglia), *Dhcr24* (astrocytes), and *Psen1* (neurons). The DEG *Htra1* (microglia, neurons, and astrocytes) formed connections with both dementia and cerebrovascular diseases. Furthermore, DEGs of NVU cells with connections to all the diseases in common amongst them were *Ptk28* (microglia), *ApoE* (astrocytes), *Pparg* (neurons), and *Vegfa* and *App* (neurons and astrocytes). Taken together, it can be considered that obesity, by modulating cell-specific expression of genes in endothelial cells and other NVU cell types, contributes to gene alterations in a pattern associated with dementia and cerebrovascular diseases.

### 2.7. BBB Permeability Changes in the Brain with Obesity

**Figure 7:**
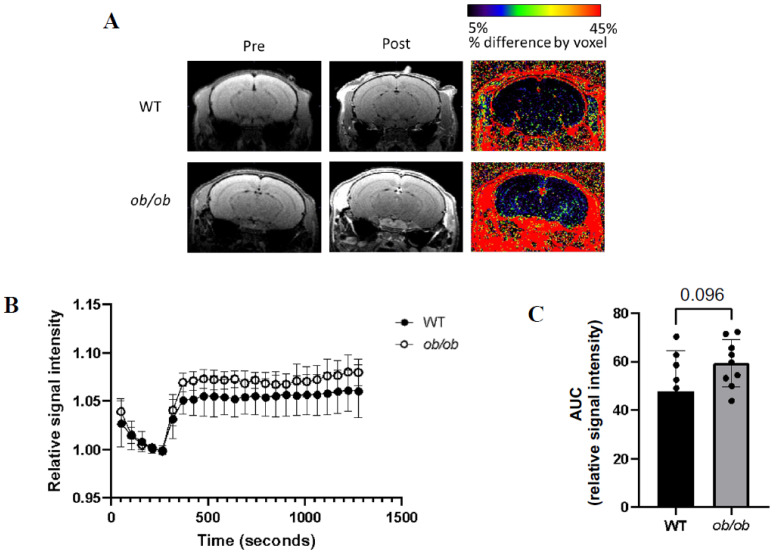
**Obesity predisposes to increased blood–brain barrier permeability.** (**A**) Representative magnetic resonance imaging (MRI) images of the brain pre- and post-gadolinium (Gd) infusion for *ob*/*ob* and WT mice and percent difference in intensity. (**B**) Dynamic contrast enhanced (DCE) plots showing relative signal intensity (y-axis) over time (x-axis). (**C**) Area under the cure (AUC) of the DCE plot for *ob*/*ob* and WT mice (*n* = 8–9/genotype).

One of the major cellular functions identified by our snRNAseq data was vascular permeability, a key cellular process underlying development of neurovascular diseases, including dementia. We, therefore, assessed BBB permeability using gadolinium (Gd) enhanced MRI of the whole brain and hippocampus. Figure 7A shows the signal intensity before and after Gd infusion and the percent difference in signal intensity in the brains of *ob*/*ob* and WT mice. DCE plots revealed slightly higher relative signal intensity in the hippocampus of *ob*/*ob* mice (Figure 7B) and a trend (*p* = 0.096) towards a larger resulting area under the curve (AUC) when compared to the hippocampus of WT mice (Figure 7C). Although not reaching statistical significance, these findings suggest that molecular changes revealed by snRNAseq may be associated with BBB dysfunction characterized by altered BBB permeability in obese mice. Longer periods of exposure of the murine hippocampus to obesity may be needed to clarify this finding.

## 3. Discussion

In this study using snRNAseq, we demonstrated obesity-induced transcriptomic changes in murine hippocampal NVU endothelial, astrocyte, microglial, and neuronal cells. Endothelial differential gene expression was cell-specific, though correlated with gene expression in the other NVU cell types, for both protein-coding and non-coding genes (lncRNAs). Obesity impacted key cellular pathways, including cell–cell interactions, the immune system, metabolism, cell signaling, and neurofunction-related pathways. DEGs were associated with neurodegenerative diseases such as dementia, Alzheimer’s, and cerebrovascular diseases. Furthermore, neuroimaging revealed a trend for increased BBB permeability in obesity. We discuss our findings in the context of the impact of obesity on endothelial cells, the relationship of endothelial cell differential gene expression to the other cells of the NVU, and key pathways modified in common for all cell types of the NVU, including cellular cross-talk.

### 3.1. Impact of Obesity on the Transcriptome of Brain Hippocampal Endothelial Cells

Obesity mostly upregulated DEGs in endothelial cells for pathways regulating endothelial permeability such as focal adhesion (Prkcb and Vegfc), adherens junction (Pard3), leukocyte transendothelial migration (Prkcb and Vcam1), and Rap1 signaling (Pard3, Prkcb, and Vegfc). It has been previously observed that protein kinase c beta (Prkcb) levels are elevated in different organs of *ob*/*ob* mice [34,35]. Increased expression of Prkcb disrupts an in vitro model of BBB in human brain microvascular endothelial cells under ischemia by regulating cell–cell junctions [36] and leads to microvascular dysfunction in murine coronary small arteries [37]. In our study, obesity also increased the expression of vascular cell adhesion molecule-1 (Vcam1), involved in leukocyte adhesion and transendothelial migration [38]. Vcam1 levels were upregulated in a murine model of vascular dementia, and inhibition of Vcam1 reduces ischemia-induced neuroinflammation and cognitive dysfunction [39]. The Par-3 Family Cell Polarity Regulator (Pard3) gene was also upregulated in endothelial cells and is expressed at the BBB and involved in the formation of adherent [40] and tight junctions [41]. Pard3 also plays a critical role in recruiting leukocytes during inflammation [42]. Vascular endothelial growth factor (Vegfc) was one of the downregulated genes in endothelial cells in *ob*/*ob* mice. Vegfc promotes angiogenesis [43], and its levels are reduced in persons with coronary heart disease [44]. Taken together, these results show that obesity modulates the expression of genes with resultant dysfunction of endothelial cell junctions, increased leukocyte infiltration, and BBB permeability, changes that are known to be associated with cognitive impairment and vascular dementia. To provide a comprehensive understanding of the impact of obesity on neurological health, we assessed behavior (by open field test) and cognition (by Y-maze and Morris water maze) functions of *ob*/*ob* and WT male mice, and recently published these findings [45]. Even though we did not observe a significant impact of obesity in these parameters, previous studies using different mouse models of obesity or ages of mice showed that obesity contributes to cognitive dysfunction [46].

### 3.2. Relationship of Endothelial Cells to the Other Cells of the NVU

Endothelial cells are not only involved in the regulation of the BBB permeability but can release mediators that affect other cells of the NVU, including microglia, astrocytes, and neurons [47]. Microglia are resident brain macrophages that play a role in the immune functions of the central nervous system. In animal models of obesity, there is a higher presence of activated microglia that is characterized by an increased production of proinflammatory cytokines [48]. Cytokines have a ubiquitous role in neurodegenerative diseases that proceed through BBB functional abnormalities [49]. Our snRNAseq data showed that obesity modulated expression of genes in microglia that are involved in cell–cell adhesion, cell signaling, and regulation of inflammation and immune response (31 DEGs), including chemokine signaling and IL-2 signaling. Moreover, changes in the expression of genes in microglia were correlated with several identified DEGs in endothelial cells, including *Fkbp5*, which impacts microglia polarization [50]; *Picalm,* which has been linked with AD development and regulation of the immune system [51]; and *Mef2a,* which has been associated with AD pathogenesis [52]. These observations suggest an increase in inflammatory response in microglia with obesity and a cross-talk between endothelial and microglial cell types in a pattern that could contribute to cognitive dysfunction.

Astrocytes are a major glial cell type in the central nervous system and are essential for maintaining the neuronal environment, neurotransmitter recycling from the synaptic cleft, maintenance of the BBB, and regulation of energy homeostasis [53]. We showed that obesity exerts a significant impact on the astrocyte genomic profile by affecting the expression of over 1000 genes, both protein-coding genes and lncRNAs, involved in the regulation of processes such as integrin-mediated cell adhesion, cell–cell adhesions, axon guidance, inflammatory cell signaling, and glutamatergic or GABAergic synapses. Astrocytes form endfeet, adhere to endothelial cells, and are important for endothelial cell permeability and cell–cell junctions that form via actin cytoskeleton reorganization, axon guidance, and focal adhesion kinase [54]. To the best of our knowledge, no studies have reported genomic changes in astrocytes with obesity in vivo. Interestingly, we also show that nearly 40 DEGs in astrocytes correlated with DEGs in endothelial cells, suggesting potential cross-talk between these two cell types. Among the correlated genes was cadherin, a cell adhesion molecule that plays a crucial role in the adhesion of astrocytes to endothelial cells via integrins and therefore maintains BBB integrity [55]. Hence, the genomic analyses suggest that obesity modulates interactions between astrocyte endfeet and endothelial cells, which potentially results in increased endothelial cell permeability.

Another important cell type of NVU are neurons. They play a major role in receiving and transmitting information throughout the body and form a large cellular network regulating brain functions such as memory, cognition, movement, and behavior [54]. We demonstrated that obesity exerts a significant impact on the modulation of gene expression in neurons, consistent with neurons constituting the largest group of cells in the brain. These genes were involved in processes regulating synapses, long-term potentiation, synaptic vesicles, cell signaling, and cell junctions. A few studies have suggested that other metabolic processes, such as high-fat diets, impact the global transcriptomic profile of neurons, including genes involved in similar cellular processes, like cell–cell adhesion, signal transduction, and axon guidance [56], and that these changes can be associated with Alzheimer’s disease development [57,58]. Moreover, correlation analyses between DEGs in endothelial cells and neurons in our studies identified 66 genes that correlated between endothelial and neuronal cell types. Among these genes were Klhl2, which has been shown to induce neuronal apoptosis [59], and Slc38a2, known to be associated with Alzheimer’s disease [60] and modulated by nutritional stress, like amino acid deprivation [61]. Furthermore, endothelial cells can impact neurons through the release of VEGF, which promotes neuronal migration through reorganization of the actin cytoskeleton via focal adhesion kinase [62], thereby contributing to the maintenance of neuronal function. Thus, the endothelial–neuronal cell relationship in our study indicated that obesity could impact normal neuronal signal transduction and therefore also contribute to cognitive decline in dementia.

### 3.3. Common Mechanisms of Transcriptomic Disruption by Obesity in All Cells of the NVU

**Figure 8:**
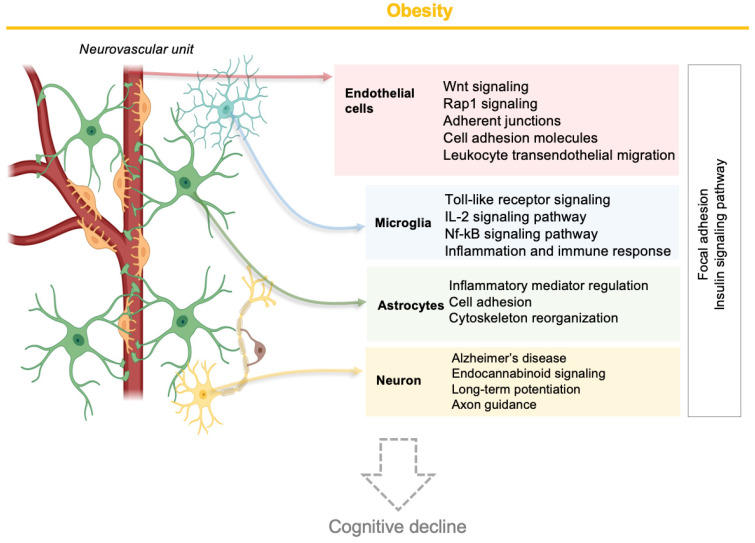
Summary schematic of the effect of obesity on the cellular functions of the hippocampal neurovascular unit and implications for cognitive decline.

Figure 8 summarizes the impact of obesity in the hippocampus on endothelial cells and their relationship to other cell types of the NVU; there were two common mechanisms of cellular transcriptomic disruption in response to obesity for all the NVU cell types, namely, focal adhesion and insulin signaling. Although little is known about the role of insulin signaling on the activity of NVU cells, impairment of insulin signaling has been identified as a mechanism in neurodegenerative diseases [63]. For example, insulin signaling in astrocytes co-regulates behavioral responses and metabolic processes via the regulation of glucose uptake across the BBB [64]. The loss of insulin signaling also leads to a reduction in dopamine release by astrocytes, affecting neuronal activity involved in cognition and mood [65]. A high-fat diet impairs insulin sensitivity in the hippocampus [66]. Furthermore, neuronal insulin signaling and insulin resistance may impact downstream signaling and synaptic plasticity, known to be impaired in neurodegenerative diseases such as Alzheimer’s disease [67]. Similarly, insulin resistance can induce activation of microglia in the hippocampus of young rats, alongside increased expression of inflammatory molecules COX-2 and IL-1β [68]. This suggests a link between neuroinflammation and insulin signaling in the hippocampus as a physiopathological mechanism underlying the connection between insulin resistance and cognitive decline. Moreover, defects in insulin signaling in microvascular endothelial cells at the BBB strongly contribute to brain insulin resistance in Alzheimer’s disease in association with β-amyloid pathology [69]. Lastly, inactivation of the insulin receptor on brain endothelial cells of the hippocampus alters the structure and increases permeability of the BBB by regulating tight junctions [70]. Therefore, disruption of insulin signaling in all cells of the NVU of mice with obesity appears to affect multiple cellular processes known to be related to mechanistic disturbance underlying dementia.

### 3.4. Cellular Cross-Talk by Obesity for Cells in the NVU

In addition to interactions between endothelial cells and other cells of the NVU, cross-talk between all four cell types needs to be taken into consideration. Astrocyte activation releases inflammatory markers and chemokines that activate microglia, which in turn release cytokines that can negatively affect neuronal activity [54]. Similarly, activated microglia release inflammatory cytokines that not only impact neuronal function but also endothelial cell permeability. Moreover, astrocytes can directly influence neuronal cells by releasing neurotoxins. In our study, we observed that obesity modulated the expression of genes coding for cytokines, cell–cell adhesion proteins, and inflammatory mediators. For example, expression of Per2 was decreased in astrocytes and can prevent neurotoxicity [54,71], therefore suggesting induction of neurotoxicity in neuronal cells in the condition of obesity. Also, expression of Cx3cl1 in astrocytes was downregulated in our study. It codes for the protein involved in the inactivation of microglia, regulation of immune homeostasis, and can counteract neuroinflammation [72]. Suggesting that obesity could lead to activation of microglia and an increase in neuroinflammation through reduced Cx3cl1 expression. Obesity also impacted the expression of Vegf in astrocytes that has been reported to be associated with neurotoxicity and increased endothelial cell permeability, as well as the expression of cytokines that impact endothelial cell–cell adhesion and BBB permeability [73].

### 3.5. Limitations

Although our study presents several strengths, such as simultaneous in-depth genomic analyses of cell types of the NVU, there are a few limitations. Several mouse models of obesity exist, and we chose the most commonly used and well characterized, *ob*/*ob* mice, homozygous for a mutation in the leptin gene. These mice present the phenotype of obesity, including glucose intolerance and insulin resistance; therefore, it is difficult to separate the effects of obesity from those of a type 2 diabetes mellitus phenotype. It should be noted, however, that in humans, obesity is generally accompanied with diabetes, and thus, the metabolic findings obtained using the *ob*/*ob* model parallel those of human obesity. Also, in our bioinformatic analyses, we grouped different types of neuronal cells together. As different neuronal cell types present slightly different biological functions, it could be possible that they also respond differentially to obesity. Subtyping and bioinformatic analyses of neuronal subtypes were beyond the scope of our studies. The association between differentially expressed genes and neurodegeneration needs to be taken with caution, as has previously been discussed in the context of cancer research [74]. However, to mitigate against this risk in identifying potential neurodegenerative diseases associated with the observed genomic changes, we used a toxicogenomics database that utilized manually curated literature-based interactions that minimize the risk of false positive interactions. Moreover, our MRI analyses suggested a tendency towards an increase in BBB permeability in *ob*/*ob* mice. The lack of statistical significance of the MRI data was probably due to the small number of mice used in the analyses, possibly the relatively short exposure (compared to lifetime) of mice to obesity, and the sensitivity of the imaging findings that may require a larger number of mice per group to be able to detect small changes in permeability.

## 4. Materials and Methods

Research was conducted in conformity with the Public Health Service Policy on Humane Care and Use of Laboratory Animals and reported in compliance with ARRIVE guidelines. The institutional review board of the University of California, Davis, the Institutional Animal Care and Use Committee (IACUC) approved this project protocol number 22,598 on 14 December 2021.

### 4.1. Experimental Animals

The most common murine model of obesity is the leptin-deficient *ob*/*ob* mouse [75,76]. Leptin is a key regulator of body weight, and lack of this gene results in hyperphagia and a decrease in energy expenditure, with resultant obesity by 4 weeks of age [76]. Some studies of *ob*/*ob* mice found memory deficits between 13 and 23 weeks of age [77,78,79] Previous studies using *ob*/*ob* mice demonstrate vascular consequences in this model, including reduced endothelial vasoregulation [80], blood–brain barrier (BBB) disruption, and impaired inflammatory responses following ischemia [81,82]. The NVU plays a significant role in BBB [21] and vascular dementia pathology [22]. Thus, the *ob*/*ob* murine model is ideally suited for the study of the impact of obesity in the vasculature and the NVU.

Male *ob*/*ob* (stock number 000632 and strain B6.Cg-Lep<ob>/J, Jackson Laboratories, Bar Harbor, ME, USA) and C57BL/6J wild-type control male mice (WT; Jackson Laboratories, stock 000664) (*n* = 20/genotype) were studied at 17–18 weeks of age. Animals were housed individually in duplex cages in a temperature- and humidity-controlled environment with a 12 h light/dark cycle in the University of California, Davis Mouse Biology Program. All mice were fed the AIN-93M purified diet (catalog number, TD.00102 Envigo Teklad diets, Madison, WI, USA) ad libitum for the 8-week study period. This standard purified diet is composed of 4.1% fat, 68.3% carbohydrate, and 12.4% protein (*w*/*w*). Food and water intake, as well as activity, was monitored daily by vivarium staff to ensure the wellbeing of the animals. At 18 weeks, after euthanasia by exsanguination under ketamine and xylazine anesthesia, mouse brains (*n* = 4 per genotype) were quickly removed, and the hippocampus was dissected from the left hemisphere, immediately frozen in the vapor phase of liquid nitrogen, and stored at −80 °C until use. The remaining mice were sacrificed and used for other analyses in this study, as detailed below.

### 4.2. Blood Metabolic Assays

Fasting serum glucose and glucose tolerance (Accu-Chek Aivia plus test strips, Roche, Basel, Switzerland) following an intraperitoneal injection of 2 g/kg glucose at 15, 30, 60, and 120 min were measured in blood sampled by tail slit (*n* = 16–20/genotype). Blood for the analysis of fasting insulin and total cholesterol was obtained by ventricular puncture at the time of euthanasia (*n* = 10/genotype). Insulin was measured by electrochemiluminescence (Meso Scale Discovery, Rockville, MD, USA), and total cholesterol was measured by enzymatic assay (Fisher Diagnostics, Middleton, VA, USA) in triplicate in non-pooled samples.

### 4.3. Hippocampal Single Nuclei RNA Sequencing

Thousands of single nuclei transcriptomes from the hippocampal brains of *ob*/*ob* and control wild-type mice (*n* = 4 brains/genotype as per above) were profiled using Parse Evercode single nuclei technology, as previously described [32]. The number of replicates was chosen to balance the cost and precision [83]. Up to 3,000,000 hippocampal nuclei were isolated, fixed, and counted using the Parse Biosciences Nuclei Fixation Kit (Catalog # SB1003, Parse Biosciences, Seattle, WA, USA). The nuclei suspension was preserved as recommended by the manufacturer’s instructions and kept at −80 °C until library preparation. Barcoded single-cell libraries were prepared from fixed single nuclei suspensions using the Evercode Whole Transcriptome Mega kit (Catalog # EC-W01050, Parse Biosciences, Seattle, WA, USA) by the UC Davis DNA Technologies and Expression Analysis Core. For the barcoding and library preparation, a maximum of 1 million cells can be sequenced for all samples studied. Given the number of samples and experimental groups used in this study, tens of thousands of nuclei/samples were barcoded and had a library prepared. We used an Agilent Bioanalyzer (Agilent Technologies, Santa Clara, CA, USA) to check the cDNA trace to assess the RNA quality of the nuclei samples. The cDNA traces of our samples had the correct size distribution with minimum small peaks indicative of RNA degradation. The cDNA and library fragment size distribution were verified on a Bioanalyzer 2100 (Agilent) and TapeStation (Agilent), respectively. The libraries were quantified by fluorometry on a Qubit instrument (LifeTechnologies, Carlsbad, CA, USA) and by qPCR with a Kapa Library Quant kit (Kapa Biosystems-Roche, Wilmington, MA, USA) prior to sequencing. The libraries were sequenced on a NovaSeq 6000 sequencer (Illumina, San Diego, CA, USA) with paired-end 100 bp reads. The sequencing generated approximately 35,000 reads per cell. We used 2 sublibraries for sequencing, which provided sequences for about 3500 nuclei for each sample. Thus, the expression of each gene for each of our 4 samples per genotype is the average expression from a very large number, that is, thousands, of nuclei. We assessed the nuclei and RNA quality in the snRNAseq data using the following metrics: We determined the Q30 in the barcodes, which is the most important metric for read quality, and included the fraction of spot barcode bases with a Q-score greater than or equal to 30 and excluded very low quality/no-call (Q lesser than or equal to 2) bases from the denominator. Sequencing characteristics and alignment metrics can be found in Supplementary Data S3.

Processing of the snRNA seq data was performed with assistance of the UC Davis Bioinformatics Core. Raw sequencing data for two sublibraries were preprocessed and combined using Parse Biosciences’ split-pipe pipeline (v0.9.6p). We assessed the quality of snRNAseq data using the nFeature_RNA plot that showed the number of detected genes in every cell, the nCount_RNA plot that showed the number of detected Unique Molecular Identifiers (UMIs) in each cell, and the percent.mito plot that revealed the percentage of mitochondrial genes in each cell (Figure S8). Expression matrices were imported into Seurat [84] for downstream analyses. Filters were applied to retain cells that had 500–10,000 genes expressed with 1000–50,000 UMIs detected, and the fraction of mitochondrial reads was less than 5%. After filtering, the cells classified as doublets were removed using DoubletFinder [85]. The remaining data for all samples were merged in Seurat, normalized using “LogNormalize” mode, and scaled to regress out cell cycle effect and sequencing depth (using the number of UMI as a proxy). The first 50 principal components were used to cluster the cells using the “FindClusters” function, using a shared nearest neighbor modularity optimization-based clustering algorithm, at resolution level 2 in Seurat and generate UMAP (Uniform Manifold Approximation and Projection) embeddings. Cell types were identified using R package ScType [86] with brain and immune system markers (Supplementary Data S4). UMAP in snRNA-seq analysis reduces high-dimensional gene expression data to 2D or 3D, preserving data structure. This aids in visualizing cell clusters, identifying distinct cell types, and interpreting complex relationships, thereby enhancing the understanding of cellular diversity and interactions in the dataset. From the identified cell types in the entire hippocampus, relative changes in the differentially expressed genes (DEGs) of the neurovascular unit (NVU)—endothelial cells, microglial cells, astrocytes, and neurons—were generated by comparing gene expression levels of *ob*/*ob* to WT mice. Moreover, in order to better assess the biological significance of the DEGs, we did not consider DEGs individually but rather after gene ontology and functional pathway analysis.

### 4.4. Brain Magnetic Resonance Imaging (MRI)

MRI scans of *n* = 8–9 brains per genotype were performed at the UC Davis Center for Molecular and Genomic Imaging (CMGI) using a Bruker Biospec 70/30 (7T) preclinical MR scanner (Bruker, Billerica, MA, USA). Images were acquired on anesthetized animals, reconstructed, and parametric maps generated using Paravision 6. BBB permeability was investigated using 1 mmol/kg of Gadolinium [87]. Infusion rates were adjusted to animal weight to generate a Dynamic Contrast Enhanced (DCE) scan [88]. Pre- and post-T1 weighted Gd scans and a post-AT2 weighted anatomical scan were also acquired. Average enhancement was calculated over the entire hippocampal region. Additional experimental details for our murine MRI imaging studies have been previously reported [32].

### 4.5. Data and Statistical Analysis

The ggplot2 R Package tool was used to create visualizations, specifically to display the log2 fold changes and the number of differentially expressed genes (DEGs) with an FDR-adjusted *p*-value less than 0.06 [89]. FDR (False Discovery Rate) is a statistical method used to correct for multiple comparisons in hypothesis testing. It adjusts *p*-values to control the expected proportion of false positives among the significant results. FDR helps reduce the likelihood of incorrectly identifying results as statistically significant due to chance.

Sparse Partial Least Squares Discriminant Analysis (sPLS-DA) is employed in snRNA-seq data analysis to pinpoint genes that distinguish between cell types. It builds on Partial Least Squares (PLS), a statistical technique that models relationships between predictors and responses by identifying orthogonal components that maximize covariance between these variables. PLS is particularly effective for high-dimensional or collinear data, reducing dimensionality while retaining essential information. sPLS-DA combines PLS with variable selection to focus on the most relevant features, enhancing classification accuracy and interpretability. This approach identifies key biomarkers, aiding in the classification of cells and revealing cell type differences in complex single-nucleus RNA sequencing datasets. A heatmap representing gene expression levels uses color gradients to depict the abundance of gene expression across samples. Darker or more intense colors often indicate higher expression levels, while lighter colors represent lower expression. This visualization helps identify patterns and differences in gene expression across various conditions or samples. The MetaboAnalyst v6.0 platform was used for statistical analysis and visualization, including sPLS-DA, Variable Importance in Projection (VIP) scores, and heat maps [90].

The Galaxy Platform was used to generate volcano plots, which visually represent the magnitude and significance of DEGs [91]. An interactive Venn tool was employed to create Venn diagrams, which show the overlap between different sets of DEGs [92]. GeneTrail Online Database was utilized to perform overrepresentation analysis and identify significant cellular pathways related to DEGs and long non-coding RNAs (LncRNAs). It adjusted for multiple testing using the Benjamini–Hochberg method with an FDR-adjusted *p*-value threshold of less than 0.05 [93,94].

The Srplot tool was used to generate various plots, including Gene Ontology (GO) plots, pathway dot plots, heat maps, and correlation plots, to visualize the functional and pathway-related aspects of the data [95]. GO analysis categorizes gene functions into biological processes, molecular functions, and cellular components. It helps identify gene functions and relationships in large datasets by using standardized terms and hierarchical structures, enhancing understanding of gene roles and interactions in various biological contexts.

Enrichr Webtool was used to identify potential transcription factors that might regulate the expression of the identified DEGs, with an adjusted *p*-value cut-off of less than 0.05 [96,97,98]. LncRRIsearch [99] and Rtools CBRC [100] were used to identify the target genes of differentially expressed LncRNAs.

Statistics were performed using Graphpad Prism 10. In order to identify outliers, the ROUT (Q-1%) test was applied to all of the following study data sets: bodyweight, glucose, GTT AUC, insulin, total cholesterol, and MRI DCE AUC. No outliers were found, and therefore no datapoints were removed. If the data were normally distributed, as determined by the Kolmogorov–Smirnov test for normality, an F-test was used to compare variances and choose the appropriate *t*-test. Groups were compared by a Student’s *t*-test if the equal variance assumption was met or compared by a *t*-test with Welch’s correction if the equal variance assumption was not met. If the data were not normally distributed, groups were compared by the two-sample Kolmogorov–Smirnov test.

## 5. Conclusions and Future Directions

Our study showed NVU cell type-specific transcriptome changes with obesity in the murine hippocampus. We demonstrated that obesity impacts brain endothelial cell gene expression, particularly genes associated with endothelial permeability, that may impact expression of genes in other cells of the neurovascular unit, a hypothesis that could be corroborated with an observed tendency towards an increase in BBB permeability. Our study revealed that the murine NVU cells respond to obesity in a cell-specific manner, pointing out the importance of simultaneous analyses of all cell types rather than one cell type analysis. Functional analyses showed that the differentially expressed genes identified regulate interactions between cell types and inflammation, changes that are associated with the development of neurodegenerative diseases like Alzheimer’s disease. Identifying the obesity-associated gene expression changes, both cell-specific and common to NVU cell types, and the cellular processes implicated, form the basis for further studies to build upon and elucidate mechanisms of cognitive dysfunction in obesity.

Future research to strengthen the findings of our study and experiments to validate the demonstrated differential expression observed in obesity and its relevance to specific cell types could include techniques such as in situ hybridization, confocal microscopy, and validation of specific genes identified in the present study (such as *Nr1d1*, *Slc39a13*, *Ddc*, *Tppp*, *Stat5b*, *Fam214a*, *Tnrc6b*, and *Muc6*). Moreover, future research should consider a broader range of ages to capture the progression of metabolic and neurological changes over time courses, and we are planning studies in aged *ob*/*ob* mice. In addition, future studies should include female mice to explore potential sex-specific differences, and we are preparing a separate manuscript for publication that will detail sex differences in the single nuclei response of the hippocampus to obesity. The influence of environmental factors and diet composition could also be addressed and could be standardized or varied in future studies to better isolate the specific effects of obesity. Finally, investigating the behavioral and cognitive consequences of the observed molecular changes could provide a more comprehensive understanding of the impact of obesity on neurological health, and we have indeed performed these studies and reported on this study [45].

## Data Availability

The snRNA-seq data reported in this paper have been deposited in the National Center for Biotechnology Information Gene Expression Omnibus database (GSE262249).

## Supplementary Materials

The following supporting information can be downloaded at: https://www.mdpi.com/article/10.3390/ijms252011169/s1.
