# Supplementary material for: Single Nuclei Transcriptomics Reveals Obesity-Induced Endothelial and Neurovascular Dysfunction: Implications for Cognitive Decline
Source: Int J Mol Sci. 2024 Oct 17;25(20):11169. doi: 10.3390/ijms252011169 (PMC11508525; doi:10.3390/ijms252011169)
Supplement: Supplementary file 1 [file ijms-25-11169-s001.zip › ijms-3165543-supplementary.pdf]

Supplementary Information

This PDF file includes:

Supplementary Tables S1 to S2

Supplementary Figures S1 to S8

Table S1. Fasting serum lipid, insulin, and glucose tolerance test (GTT) of *ob/ob* and WT mice at 17-18 weeks of age.

Serum parameters	WT	*ob/ob*
Body weight (g)	29.5 ± 2.7	51.5 ± 3.3*
Total cholesterol (mg/dL)	124.1 ± 35.9	279.1 ± 25.9*
Insulin (pg/mL)	229.1 ± 131.7	3311 ± 1625*
Glucose (mg/dL)	157.4 ± 32.6	175.6 ± 41.6
GTT AUC (mg*min/dL)	29500 ± 5380	43307 ± 8706*

Data represented as mean ± standard deviation, * $p<0.05$ compared to WT mice.

n=19 (Body weight); n=10/group (Total cholesterol, Insulin); n=16-20 (Glucose and GTT AUC).

Table S2. Different cell types in the hippocampus of *ob/ob* and WT male mice.

Cell types	Mean number of cells	
	ob/ob	**WT**
Endothelial cells	65	68
Microglial cells	69	69
Astrocytes	582	476
Neurons	2566	2218
Classical Monocytes	122	81
Macrophages	99	81
Mast cells	80	40
Neural Progenitor cells	71	37
Neural stem cells	29	30
Neuroblasts	32	27
Non myelinating Schwann cells	31	22
Oligodendrocyte precursor cells	209	376
Oligodendrocytes	390	375
Tanycytes	176	88

Cells of the Neurovascular unit (NVU) are highlighted in green. ScType database used to identify different cell types did not have markers to identify pericytes.

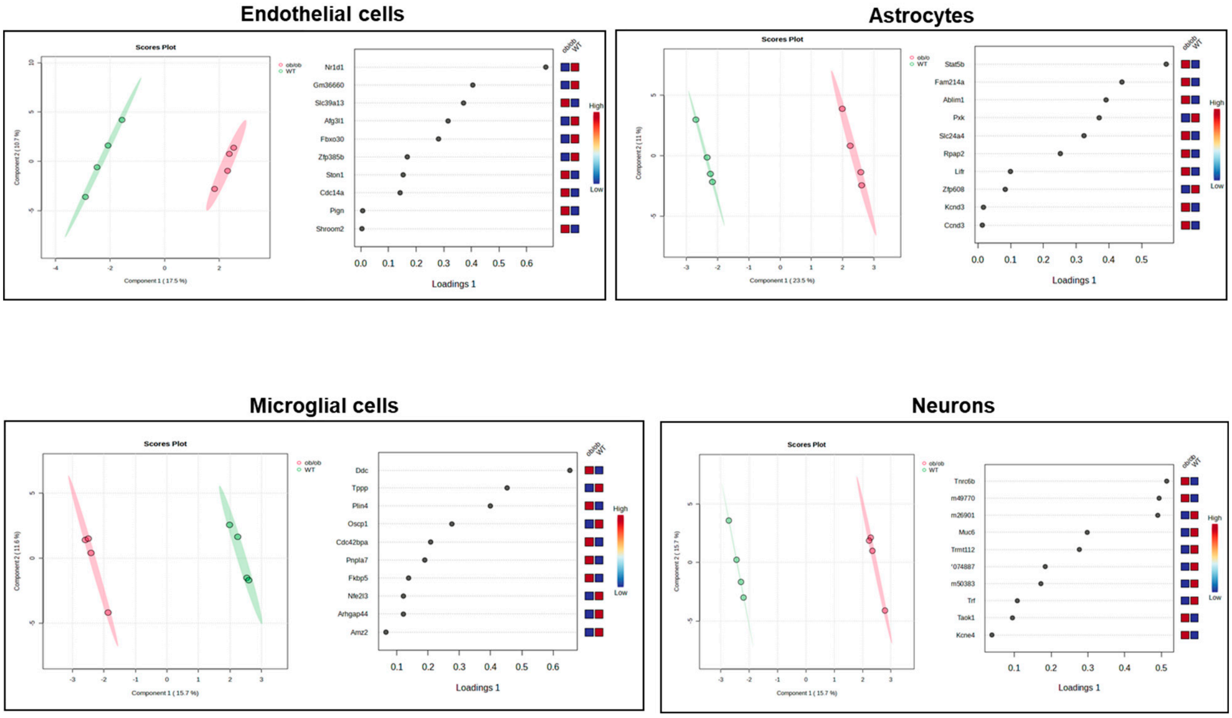

Figure S1. sPLS-DA plots and VIP scores of Neurovascular unit. Sparse Partial Least Squares Discriminant Analysis (sPLS-DA) shows normalized global gene expression profiles of hippocampal neurovascular unit (NVU) cell types: endothelial cells, astrocytes, microglial cells, and neurons in *ob/ob* mice (red circles) and WT mice (green circles), n=4 samples per genotype. Variable importance projection (VIP) scores show top 10 genes driving the sPLS-DA separation.

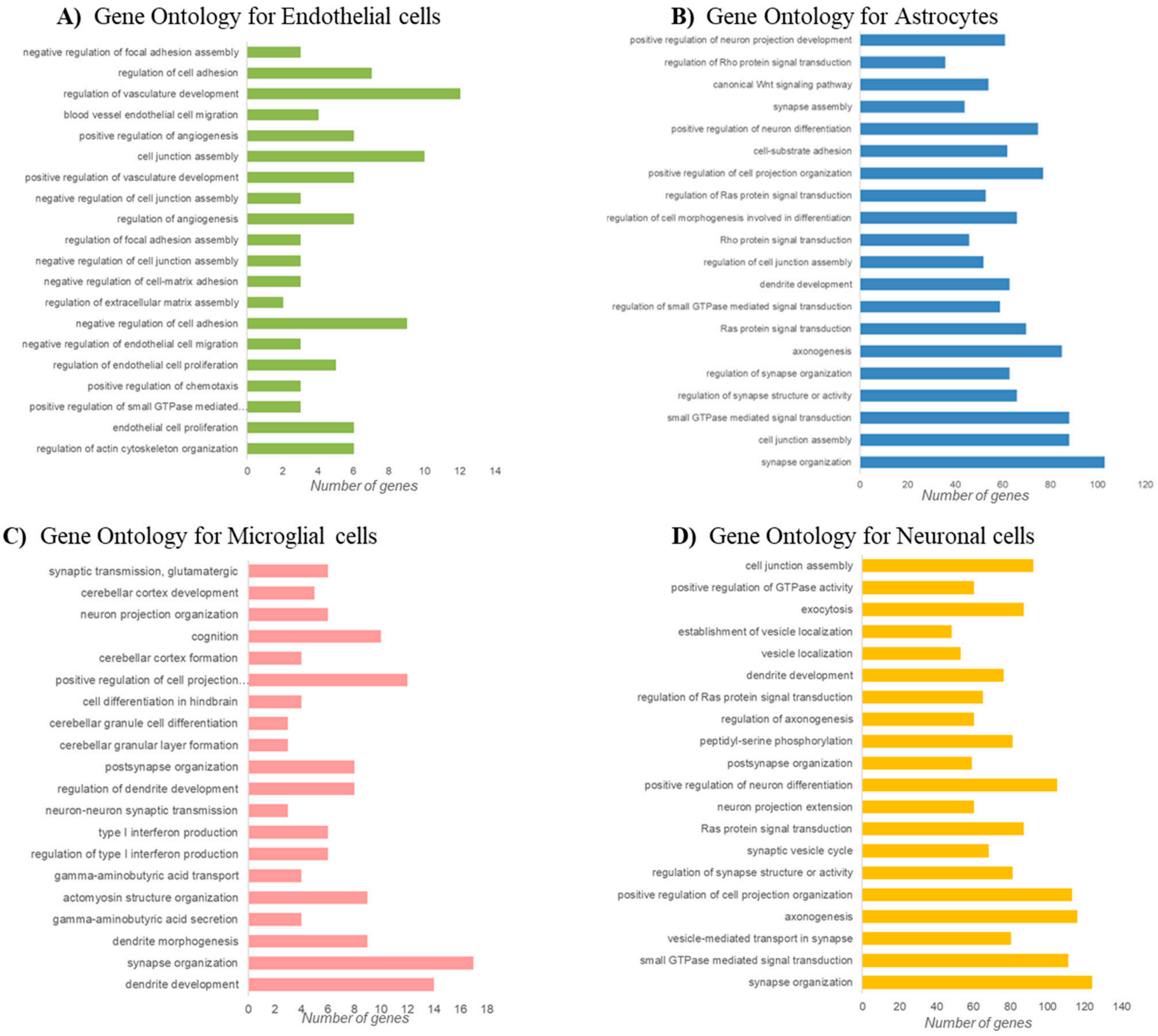

Figure S2. Gene ontology analyses of DEGs of the NVU cell types modulated by obesity reveal functional changes related to cell-cell interactions, cognition, and synapses. Gene ontology analysis of differentially expressed genes identified significantly over-represented Biological Processes in **A**) endothelial cells; **B**) astrocytes; **C**) microglial cells, and **D**) neuronal cells.

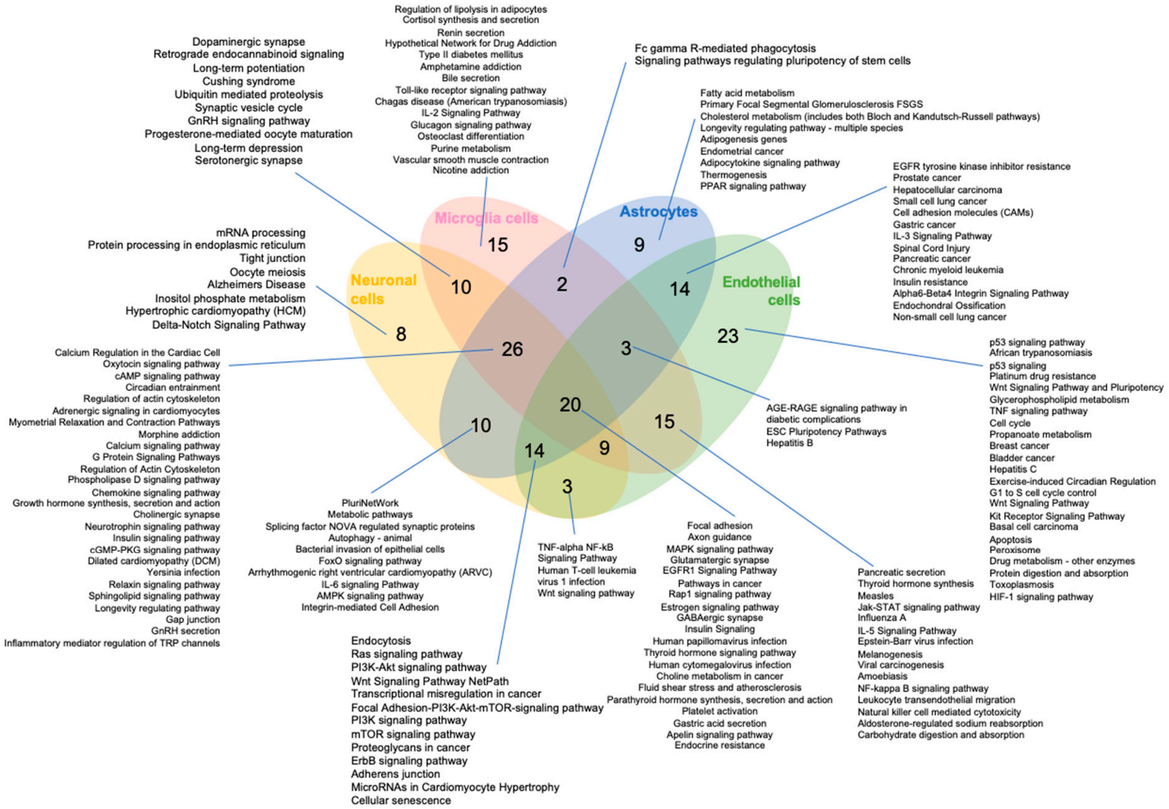

Figure S3. Comparison of significant cellular pathways of the 4 cell types of Neurovascular unit modulated by obesity. Venn diagram reveals significant cellular pathways (FDR p value <0.05) regulated by differentially expressed genes in the hippocampal cells of the NVU (endothelial cells, astrocytes, microglial cells, and neurons) of ob/ob mice compared to WT mice.

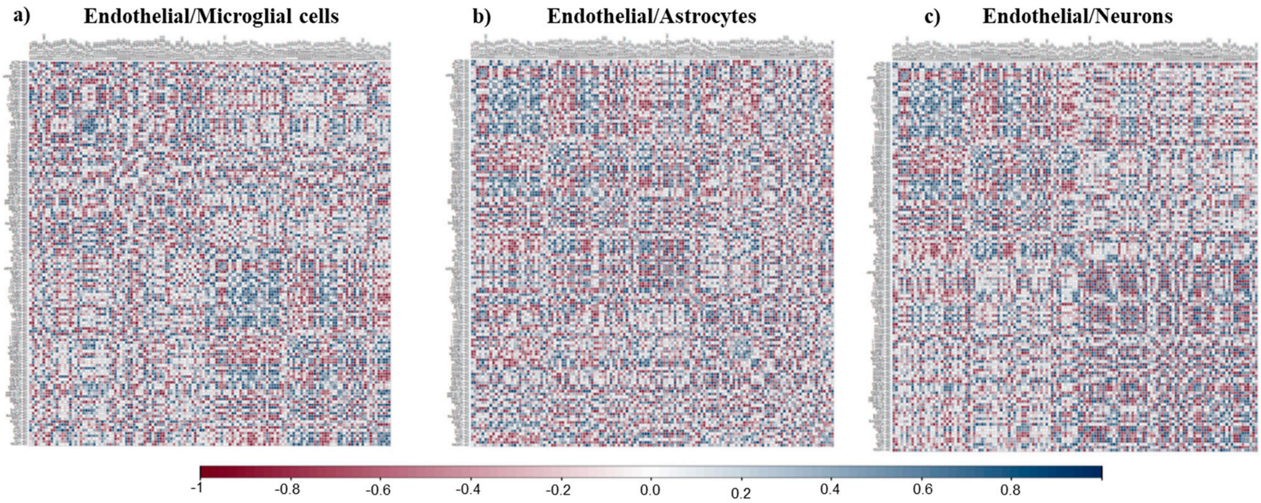

Figure S4. Correlation analyses of differentially expressed genes of neurovascular unit cell types altered by obesity in the focal adhesion pathway. Gene-gene correlation matrices of genes identified as differentially expressed in endothelial cells and microglia cells, endothelial cells and astrocytes, and endothelial cells and neuronal cells involved in the focal adhesion pathway. For each gene-gene correlation, positive correlation is presented in blue and negative correlation in red; significant correlations are presented with "*".

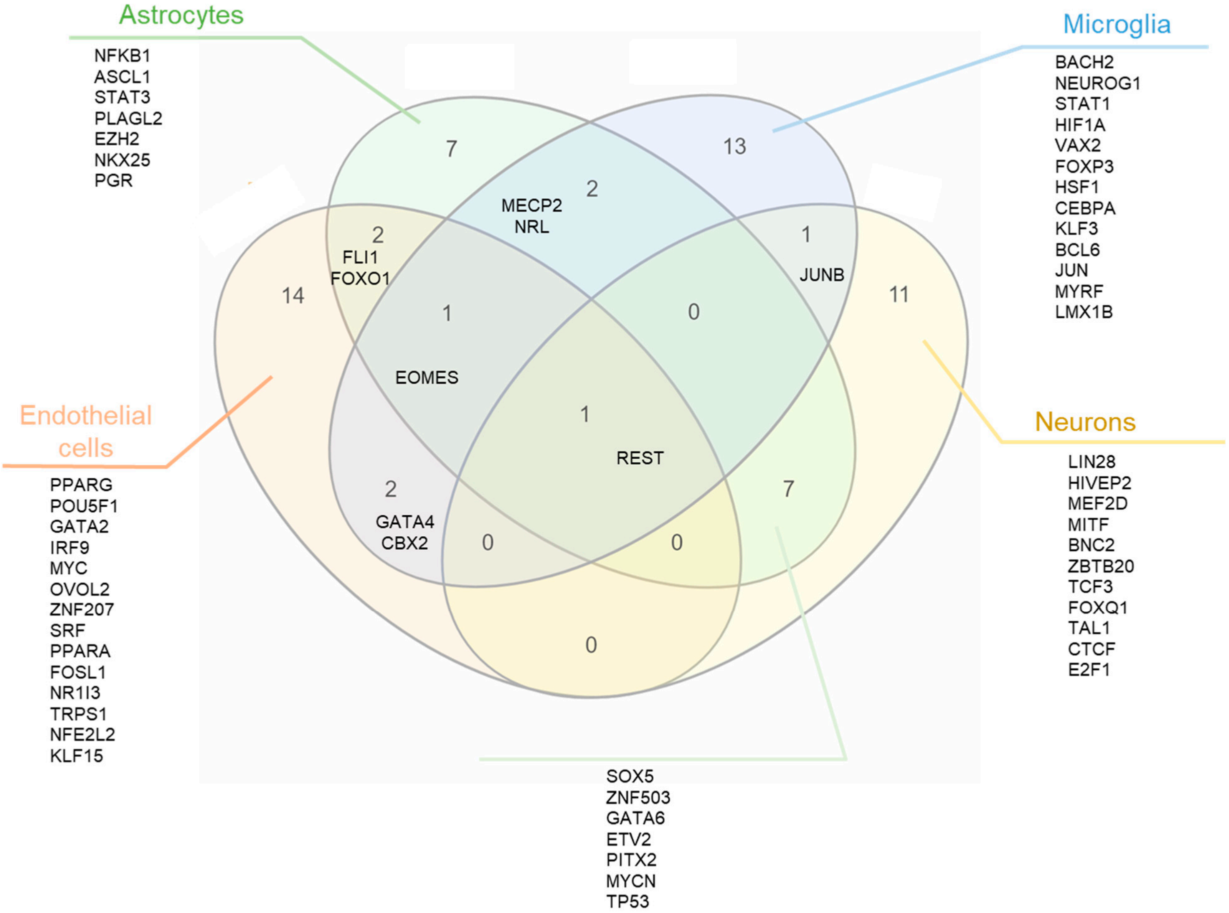

Figure S5. Comparison of top 20 transcription factors modulated by obesity in the NVU cell types. Venn diagram shows top 20 significant ($p<0.05$) transcriptional factors regulating the differentially expressed genes of hippocampal neurovascular unit cell types (endothelial cells, astrocytes, microglia, and neurons) in the *ob/ob* mice compared to WT mice.

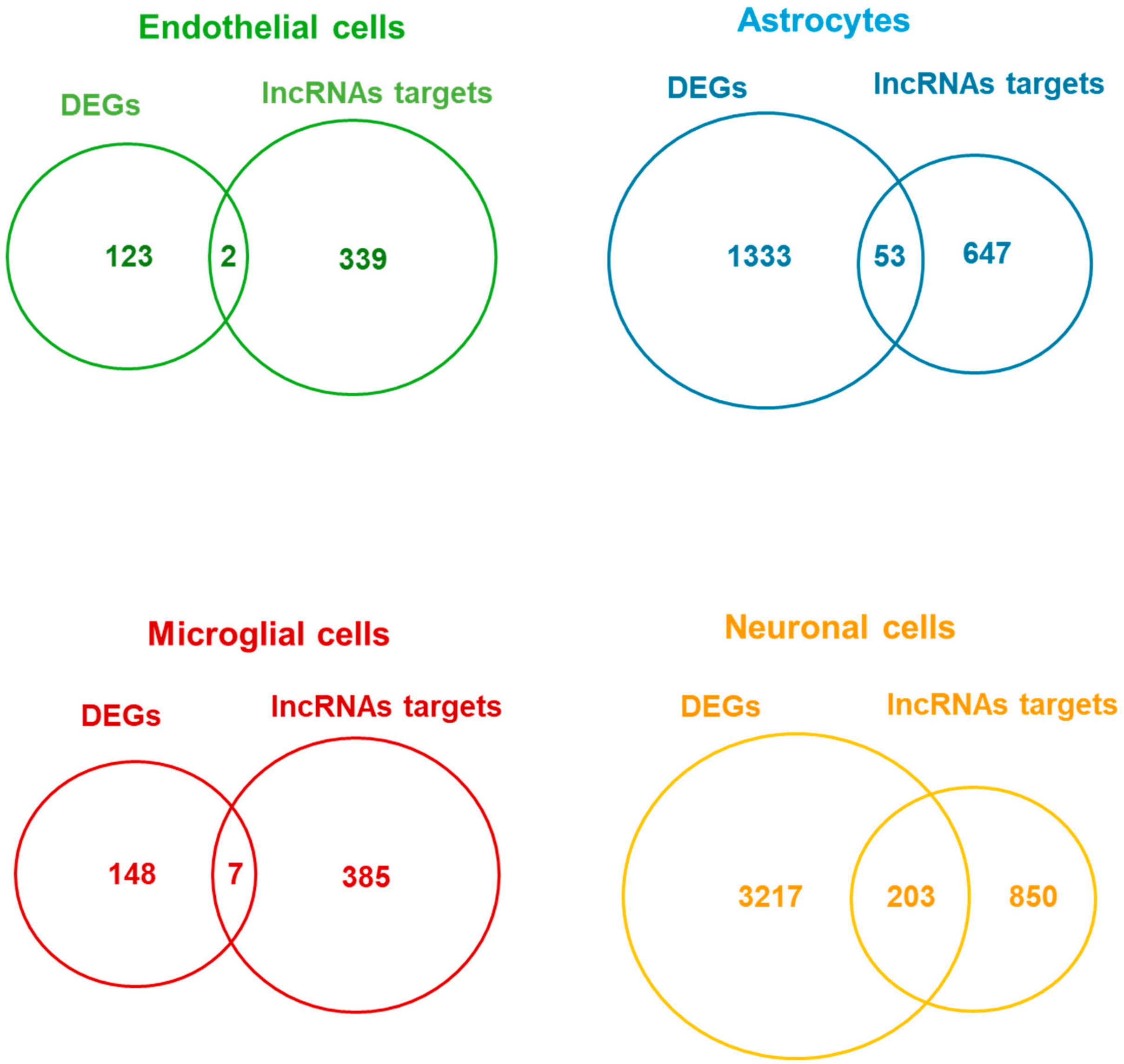

Figure S6. Comparison of differentially expressed genes and targets of top 20 long noncoding RNAs altered by obesity in the neuro vascular unit. Venn diagram of differentially expressed genes (DEGs) and targets of top 20 differentially expressed long noncoding RNAs (lncRNAs) modulated by obesity in the four hippocampal neurovascular unit cell types (endothelial cells, astrocytes, microglial cells, and neuronal cells).

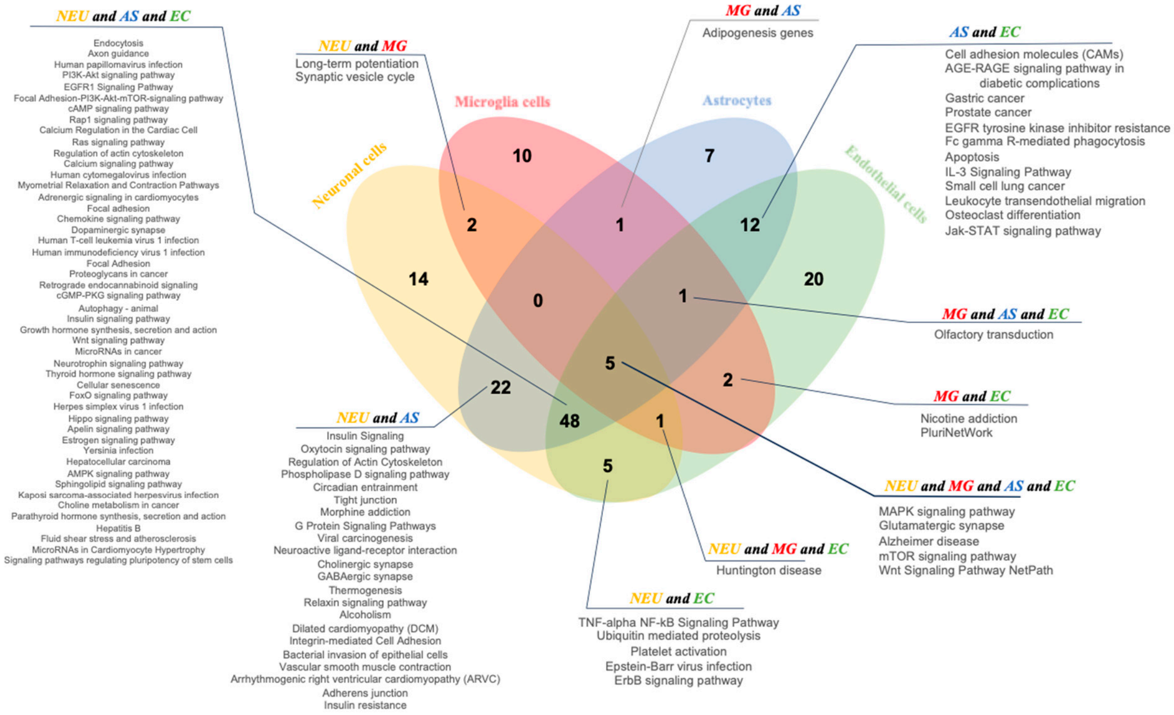

Figure S7. Comparison of significant cellular pathways of the targets of differentially expressed top 20 lncRNAs modulated by obesity in the NVU. Venn diagram reveals significant cellular pathways (FDR p value <0.05) regulated by targets of differentially expressed top 20 long noncoding RNAs (lncRNAs) in the hippocampal NVU cells of ob/ob mice compared to WT mice. Endothelial cells (EC), astrocytes (AS), microglia (MG), and neurons (Neu).

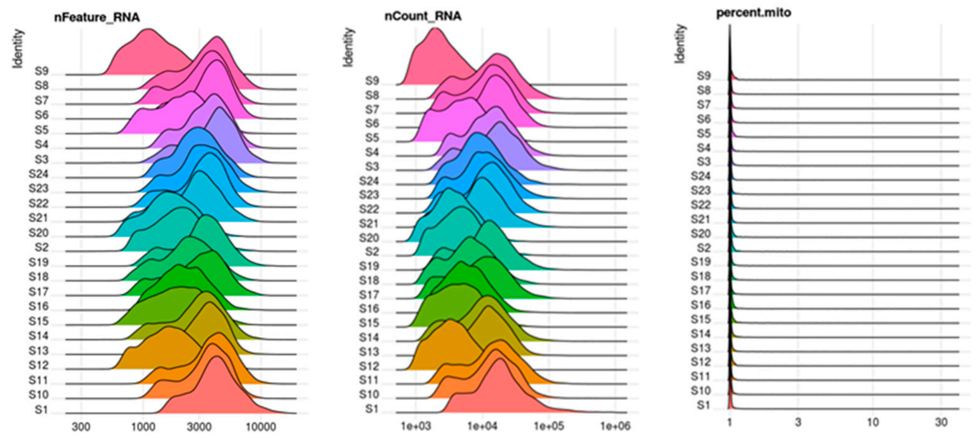

Figure S8. snRNAseq data quality assessment plots. The nFeature_RNA plot showed the number of detected genes in each cell. The nCount_RNA plot showed the number of detected Unique Molecular Identifiers (UMIs) in every cell. The percent.mito plot revealed the percentage of mitochondrial genes in each cell. The *ob/ob* samples in this study were S13,S14,S19, and S22 and WT samples in this study were S5, S11, S17, and S21.
